# Immune landscape and prognostic gene signatures in gastric cancer: implications for cachexia and clinical outcomes

**DOI:** 10.3389/fimmu.2023.1297363

**Published:** 2023-11-14

**Authors:** Xiangyu Sui, Guohao Wu

**Affiliations:** Department of General Surgery, Zhongshan Hospital of Fudan University, Shanghai, China

**Keywords:** immune landscape, gene signatures, gastric cancer, cachexia, metabolism

## Abstract

Cachexia, a debilitating condition that worsens patient outcomes, often accompanies gastric cancer, a malignancy that is prevalent worldwide. The extensive research explored the interconnected molecular and immune aspects of stomach cancer, with a particular emphasis on cachexia. By employing the GEO database, we identified genes that were expressed differently in gastric cancer patients suffering from cachexia. Following the analysis of Weighted Gene Co-expression Network (WGCNA), gene modules intricately linked to particular immune cells were revealed, indicating a significantly disrupted tumor microenvironment. A strong predictive model was developed, centered around key genes such as CAMK4, SLC37A2, and BCL11B. Surprisingly, this particular model not only showed better predictive abilities in comparison to conventional clinical factors but also exhibited a strong connection with increased infiltration of macrophages and T cells. These discoveries suggest the presence of an immune-suppressing and tumor-promoting atmosphere among individuals at a greater risk. Moreover, the utilization of Gene Set Enrichment Analysis (GSEA) established a connection between the genes linked to our risk score and vital immune-related pathways, thereby strengthening the pivotal involvement of immunity in the development of gastric cancer. To summarize, our discoveries provide a more profound comprehension of the molecular and immune mechanisms that support cachexia in gastric cancer, presenting a hopeful basis for upcoming advancements in treatment.

## Introduction

Stomach cancer, also called gastric cancer, continues to be a highly prevalent and deadly form of cancer across the globe (1). Globally, gastric cancer is identified as the third most significant contributor to cancer-related fatalities, as stated by the World Health Organization.Gastric cancer demonstrates significant geographical disparities in its epidemiology, with Eastern Asia, especially Japan and South Korea, displaying the highest occurrence rates (2). Various factors that increase the risk of developing this cancer have been identified, such as infection with Helicobacter pylori, dietary patterns, smoking, and specific genetic mutations (3). Although there have been improvements in the early identification and treatment methods, the outlook for individuals diagnosed with advanced gastric cancer continues to be unfavorable (4). Surgical removal, frequently accompanied by chemotherapy or radiotherapy, is the primary approach to treatment (5). Novel therapeutic approaches, such as targeted therapies and immunotherapies, are currently under investigation and offer promising avenues for improving patient outcomes (6). Understanding the epidemiological trends and evolving treatment landscape is crucial for devising effective strategies to combat this formidable disease.

The development of gastric cancer is influenced by both tumor cells and the surrounding microenvironment, making it a multifaceted condition (7). The immune cell infiltration is a crucial element in this microenvironment, as it has a twofold impact on both tumor progression and suppression (8). In gastric cancer, TILs, including cytotoxic T cells, helper T cells, and regulatory T cells, have been identified as highly significant (9). The balance and interaction of these immune cells can determine the tumor’s immunogenicity and the patient’s prognosis. The emergence of immunotherapy has sparked an increasing fascination with harnessing the natural defenses of the body to fight against gastric cancer (10). Clinical trials have demonstrated the potential of immune checkpoint inhibitors that target the PD-1/PD-L1 and CTLA-4 pathways, providing a ray of hope for individuals suffering from advanced illness (11).

Cachexia, commonly known as cancer cachexia in connection with malignant tumors, is a complex condition marked by the continuous depletion of skeletal muscle mass (with or without reduction in fat mass), resulting in gradual decline in physical function (12). Although cachexia holds clinical importance, its pathophysiology remains incompletely comprehended. It’s not merely a result of decreased food intake but involves a complex interplay of reduced protein synthesis, increased protein degradation, and metabolic alterations (13). In cancer patients, cachexia is particularly concerning, affecting up to 80% of advanced cancer patients (14). It has been associated with unfavorable treatment results, diminished life quality, and heightened mortality rates. Cachexia’s existence can restrict the patient’s available treatment choices and frequently decrease the effectiveness of therapies because of decreased tolerance.

Tregs, also known as regulatory T cells, are a distinct group of CD4+ T cells that have a crucial function in preserving immune balance and averting autoimmune responses (15). Tregs, which are crucial in inhibiting abnormal or exaggerated immune reactions, thus safeguarding against harm to tissues and autoimmune disorders, are distinguished by the presence of the transcription factor Foxp3 (16). In addition to their crucial function in regulating self-reactivity, Tregs have been linked to various disease states, including cancer and infectious diseases. Tregs play a central role in cancer immunotherapy research as they have the ability to suppress the immune response against tumors (17). Conversely, their suppressive function can be harnessed therapeutically to prevent graft-versus-host disease in transplant settings or to treat autoimmune disorders (18). The dual nature of Tregs, as both potential therapeutic targets and tools, underscores their significance in the field of immunology and medicine.

Our main objective is to examine the relationship between Tregs and gastric cancer, as demonstrated above. In the past few years, the advancement of bioinformatics analysis has led to extensive exploration of various approaches focused on the impact of immune-related functions on cancer. The analysis of immune cell infiltration was utilized in this study to acquire the infiltration of immune cells in a cohort of gastric cancer. Furthermore, the investigation of immune-related genes in the gastric cancer cohort was extended through the implementation of single-cell analysis.

## Methods

### Data acquisition from TCGA and GEO

Gastric cancer genomic and transcriptomic data, including RNA sequencing, clinical features, and mutation data, were procured from the TCGA database via the GDC Data Portal (https://portal.gdc.cancer.gov/). Also, microarray datasets related to gastric cancer were sourced from the GEO database (https://www.ncbi.nlm.nih.gov/geo/). GSE131835 includes 8 patients from 3 groups (cancer cachexia, cancer weight stable and control). In this work, the GSE84433 in GEO database was involved, which includes a total of 357 gastric cancer samples. In addition, the clinical features of the gastric cancer patients were also included. To ensure data consistency and reduce batch effects, both TCGA and GEO datasets underwent rigorous quality control, normalization, and batch effect adjustments. Genes were annotated using the Human Genome Organisation (HUGO) Gene Nomenclature Committee (HGNC) database, and expression values were log2-transformed for subsequent analyses.

### Analysis of immune cell data using the Weighted Gene Co-expression Network Analysis (WGCNA)

The DESeq2 package in R was used to normalize the raw expression data obtained from immune cells. Genes with counts less than 10 in more than 80% of samples were excluded. The resultant matrix was then transformed into a variance stabilizing transformation (VST) for subsequent analysis. To ensure sample homogeneity and to detect potential outliers, samples were hierarchically clustered based on Euclidean distance. Before constructing the network, any outliers that were detected were eliminated. A gene co-expression network with signed weights was built using the WGCNA package in R. The soft-thresholding strength was chosen according to the criterion of approximate scale-free structure. To reduce the impact of noise and false connections, the adjacency matrix was converted into a topological overlap matrix (TOM). The TOM underwent average linkage hierarchical clustering, resulting in the identification of gene modules through the utilization of the Dynamic Tree Cut technique. To identify modules of interest, external traits were used to correlate with modules. To identify modules significantly linked to specific immune cell characteristics, the correlation between eigengenes (representing the initial principal component of a module and serving as a representative gene expression profile) and external sample traits was examined.

### Immune cell infiltration analysis using CIBERSORT

Using the CIBERSORT web portal, the normalized expression data were uploaded for deconvolution. We chose the LM22 signature matrix, which consists of 22 unique types of immune cells (such as T cells, B cells, macrophages, and various others). To evaluate the statistical importance of the deconvolution outcomes, the algorithm was executed using the standard 1000 permutations. CIBERSORT generated a matrix of results that linked every sample to a proportion of the 22 different immune cell types. The fractions of each cell type within the sample add up to 1 (or 100%), indicating the relative proportions of each cell type in the sample.

### Utilizing GO and KEGG, conduct an analysis on pathway and functional enrichment

The `clusterProfiler` package in R was utilized to conduct the GO and KEGG analysis. The database encompasses three domains, namely Biological Process (BP), Cellular Component (CC), and Molecular Function (MF), to which the key genes were mapped. A p-value of 0.05 was considered significant for terms with an adjusted p-value.

### Prognostic model construction using Cox and LASSO regression

Univariate Cox proportional hazards regression was used to assess the individual association of each clinical and molecular variable with overall survival through initial survival analyses. Potential predictors were selected for the subsequent LASSO regression based on their p-values being less than 0.05 in the univariate analysis. In order to address multicollinearity and improve predictor selection, we utilized LASSO regression with the `glmnet` package in R. The penalty parameter was determined through 10-fold cross-validation, with the goal of minimizing the average cross-validated error. Features that had non-zero coefficients in the LASSO regression were kept for the subsequent analysis using multivariate Cox regression. The LASSO regression was used to select features for building a multivariate Cox proportional hazards regression model. The importance of every variable in the multivariate model was evaluated, and variables with a p-value less than 0.05 were kept in the ultimate model. To validate the Cox regression, the proportional hazards assumption was examined for every variable in the ultimate model.

### Validation of prognostic model accuracy

Based on the prognostic model, patients were categorized into high-risk and low-risk groups according to their calculated risk scores. Survival outcomes were evaluated using the log-rank test by plotting Kaplan-Meier survival curves for both groups. ROC curves were plotted at different time points to assess the model’s ability to discriminate over time. To measure the model’s predictive accuracy, the calculation of the area under the curve (AUC) was performed. Perfect discrimination is indicated by an AUC value of 1, whereas an AUC value of 0.5 suggests the absence of discrimination. Using the `rms` package in R, a nomogram was built by employing a multivariate Cox regression model. This nomogram visually displayed the prognostic model and enabled personalized risk estimation by summing the points assigned to each predictor variable. To evaluate the concordance between the projected and observed survival probabilities at particular time intervals, calibration plots were produced. A perfectly calibrated model would result in a 45-degree line, indicating complete concordance between predicted and observed outcomes. Both univariate and multivariate Cox regression analyses were performed to identify independent prognostic factors. The variables that were found to be statistically significant (p < 0.05) in the univariate analysis were included in the multivariate analysis. To evaluate the relationship between survival and each variable in the multivariate model, hazard ratios (HR) and 95% confidence intervals (CI) were computed, indicating the strength and direction of their association.

### The process of analyzing gene sets for enrichment using Gene Set Enrichment Analysis (GSEA)

To ascertain if pre-defined gene sets displayed statistically significant and consistent disparities between two biological conditions, we utilized Gene Set Enrichment Analysis (GSEA). The GSEA analysis was conducted utilizing the GSEA software version 4.1. For the analysis, we utilized the Molecular Signatures Database (MSigDB) collections, focusing primarily on the c2 and c5 collections. To estimate the significance level of the enrichment scores, the number of permutations was set to 1000. The sets of genes were deemed significantly enriched with a false discovery rate (FDR) below 0.25, as suggested by the creators of GSEA. GSEA calculates an enrichment score (ES) for every gene set, which indicates the extent to which a gene set is overrepresented at the top or bottom of a list of ranked genes. Afterwards, a normalized enrichment score (NES) is computed for every gene set, taking into consideration variations in gene set magnitude and associations between gene sets and the expression dataset.

### Immunohistochemistry (IHC)

To perform IHC analysis, sections embedded in paraffin were treated to remove the paraffin and restore hydration. To block the endogenous peroxidase activity, a solution of 3% hydrogen peroxide in methanol was applied for a duration of 10 minutes. The slides were heated in a citrate buffer (pH 6.0) for 20 minutes to perform antigen retrieval. To prevent non-specific binding, sections were subsequently treated with 5% BSA (bovine serum albumin) for a duration of 30 minutes. Specific markers of interest were targeted using primary antibodies, which were then left to incubate overnight at a temperature of 4°C. Following the rinsing with phosphate-buffered saline (PBS), the sections were exposed to biotinylated secondary antibodies for a duration of 30 minutes. Subsequently, the streptavidin-peroxidase complex was introduced. The resulting product was observed by utilizing 3,3’-diaminobenzidine (DAB) as a chromogen, and the sections were stained with hematoxylin for contrast. Slides were then dehydrated, cleared, and mounted. The expression patterns of the markers were examined and scored under a light microscope.

### Statistical analysis

R software was utilized for all the analyses. To determine statistical significance, a P-value of 0.05 was used. Student’s t-tests were employed for variables that exhibited a normal distribution, while the Mann-Whitney U-test was utilized for variables that did not conform to a normal distribution.

## Results

### The analysis of differential expression identified the genes that were expressed in a similar manner in the GEO database

**Figure 1:**
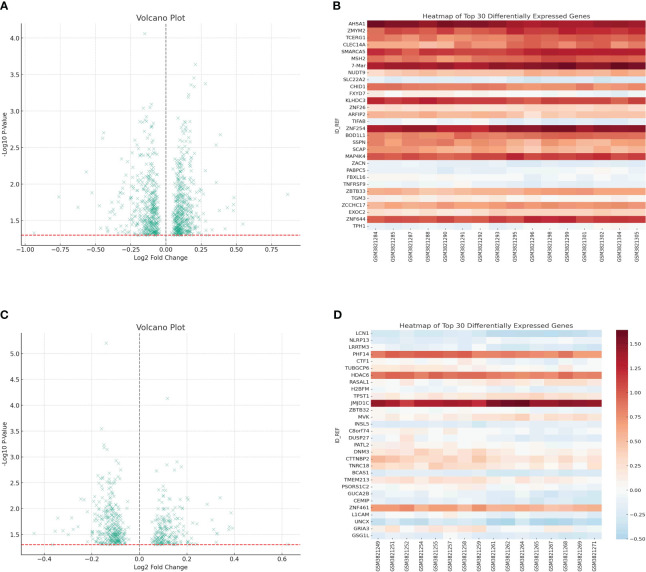
**(A)** The differentially expressed analysis in visceral adipose tissue; **(B)** The heatmap showed the top 30 differentially expressed genes in visceral adipose tissue; **(C)** The differentially expressed analysis in subcutaneous adipose tissue; **(D)** The heatmap showed the top 30 differentially expressed genes in subcutaneous adipose tissue.

The GEO database, renowned for its rich repository of gene expression datasets, presented an ideal platform to delve deeper into the gene expression profiles associated with cachexia. Leveraging this resource, our study zeroes in on dataset GSE131835, which encompasses data from a diverse cohort, providing a robust foundation for our analysis. For the purpose of investigating the crucial genes that have a significant impact on cachexia, the utilization of the geo database was incorporated to enhance the exploration of the cohort associated with cachexia. GSE131835 includes 8 patients from 3 groups (cancer cachexia, cancer weight stable and control). Furthermore, the microarray platform was employed to analyze gene expression in both visceral and subcutaneous adipose tissue of every participant. In comparison to the normal groups, a total of 951 genes exhibited altered expression with a p value less than 0.05 in relation to visceral adipose tissue (**Figures 1A, B**). In comparison to the normal groups, a total of 561 genes were identified as the differentially expressed genes in subcutaneous adipose tissue (**Figures 1C, D**).

### The single-cell RNA sequence revealed the role of immune-related cells in gastric cancer cohort

**Figure 2:**
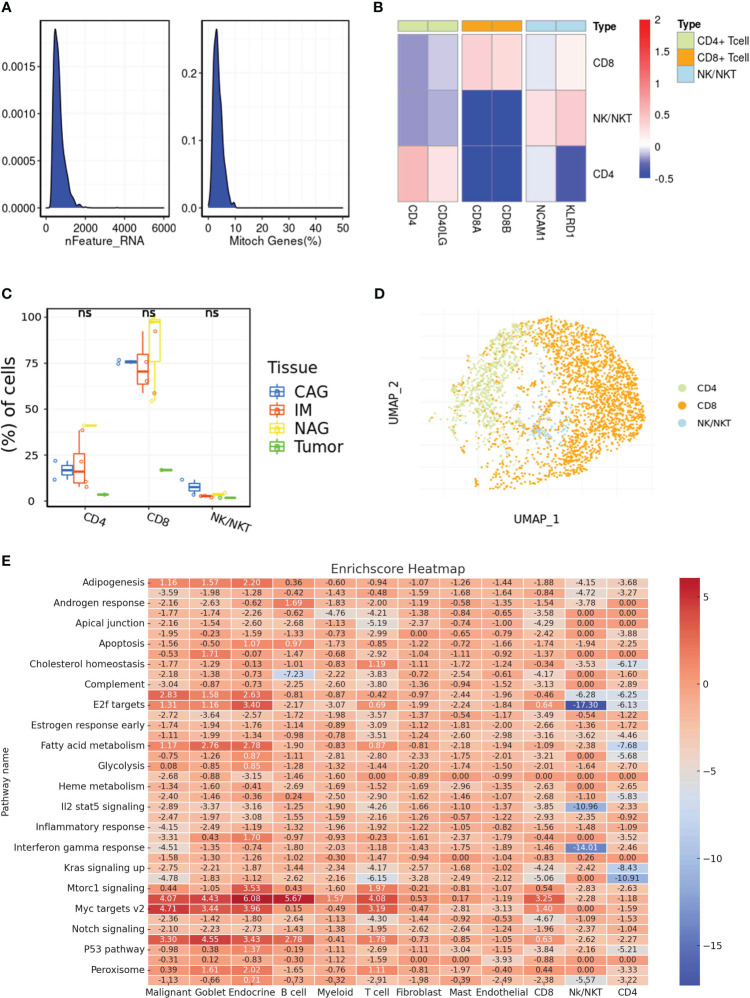
**(A)** The results of quality check in single-cell RNA sequencing; **(B)** The cell marker of different immune-related cells in single-cell RNA sequence; **(C)** The different distribution of different immune-related cells in multiple samples; **(D)** The dimensionality reduction analysis results of UAMP method; **(E)** The correlation between potential enriched pathways and immune-related cells. ns, not significant.

Unraveling the intricate interplay between immune cells and the tumor microenvironment is pivotal in understanding the multifaceted dynamics of gastric cancer progression. Especially in an era where immunotherapy is making significant strides, a granular understanding of immune cell composition and their interactions within the tumor milieu holds immense therapeutic promise. In order to explore the role of immune-related cells in gastric cancer cohort, the single-cell RNA sequence was used for the further analysis. Firstly, we single-cell RNA sequence was performed the quality check analysis (**Figure 2A**). Then, we evaluate the proportion of multiple immune-related cells in gastric cancer cohort, especially for CD4 and CD8 cells (**Figures 2B–D**). Finally, we also performed the correlation analysis to evaluate the correlation between metabolism-related pathways and immune-related cells (**Figure 2E**).

### The WGCNA analysis of the immune system revealed a gene set that is associated with various immune cells

**Figure 3:**
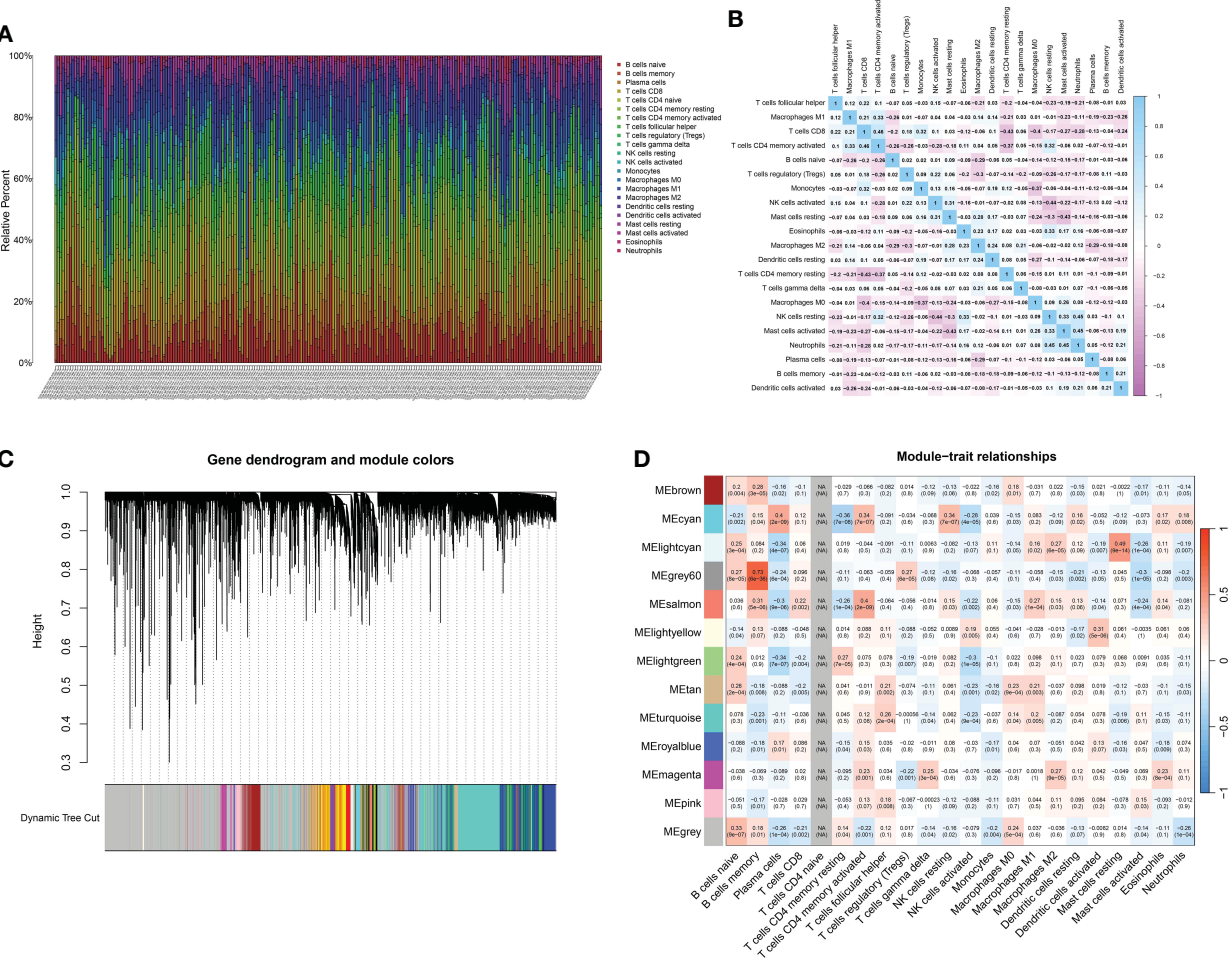
**(A)** The CIBERSOFT analysis revealed the multiple immune-related cells in renal cell carcinoma cohort; **(B)** The correlation analysis between different immune-related cells; **(C)** The dendrogram on the top showcases the hierarchical clustering of genes based on their expression patterns. Different colors underneath the dendrogram indicate gene modules identified by the analysis. Each module comprises a group of co-expressed genes with similar expression profiles; **(D)** The bar plot depicts the correlation between gene modules and external traits. Modules with strong correlations to specific traits can suggest potential biological relevance.

Immune infiltration within the tumor microenvironment has emerged as a critical determinant of tumor behavior, prognosis, and therapeutic responsiveness in many cancers, including gastric cancer. Decoding the nature, extent, and interplay of immune cells within gastric tumors holds the promise of refining our understanding of disease mechanisms, guiding prognosis, and potentially offering therapeutic targets. To assess the potential infiltration of immune cells, an analysis of immune cell infiltration was conducted in a cohort of gastric cancer patients (**Figure 3A**). The correlation analysis also indicated that the immune-associated genes exhibited possible internal correlation among themselves (**Figure 3B**). By employing WGCNA, we examined the gene expression patterns of gastric cancer specimens in order to detect gene co-expression clusters associated with immune responses (**Figure 3C**). Following the preprocessing and quality assurance of the data, we built a gene co-expression network with a soft-thresholding power of 13, chosen according to the criterion of achieving an approximate scale-free topology. There were a total of 13 different modules, with each module being represented by a distinct color. To understand the relevance of the identified modules to immune phenotypes, we correlated each module’s eigengene with immune-related traits (**Figure 3D**). The brown module demonstrated a strong positive correlation with memory B cell. In addition, the cyan module was positively associated with plasma cell. Also, the grey60 module demonstrated a strong positive correlation with memory B cell.

### Multiple analysis identifies prognostic factors

**Figure 4:**
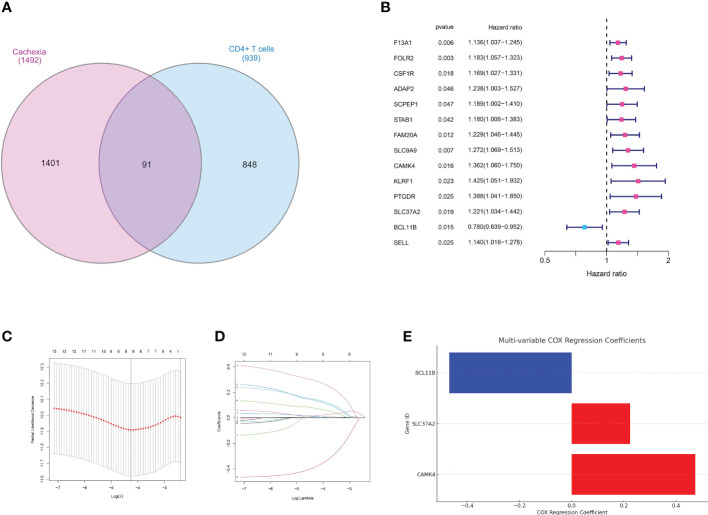
**(A)** The venn diagram demonstrated the genes that are associated with CD4+ T cells and cachexia; **(B)** The results of univariate COX regression analysis; **(C, D)** The results of LASSO regression analysis; **(E)** The results of multivariate COX regression analysis;.

In the realm of oncology, the quest for identifying genetic markers that provide insights into disease prognosis has gained immense traction. Such markers not only unravel the intricate genetic landscape of tumors but also empower clinicians with tools to predict patient outcomes, tailor treatments, and potentially identify novel therapeutic targets. Initially, the genes related to MEsalmon were utilized to investigate the genes linked to CD4 T cells. The venn diagram displayed the genes that were both upregulated and downregulated (**Figure 4A**). An initial analysis of the TCGA dataset using univariate Cox regression revealed a number of genes that showed a significant correlation with patient survival. Prominent genetic markers comprised F13A1, FOLR2, CSF1R, ADAP2, SCPEP1, STAB1, FAM20A, SLC9A9, CAMK, KLRF1, PTGDR, SLC37A2, BCL11B, and SELL (**Figure 4B**). In order to avoid model overfitting and identify the most important prognostic genes, LASSO regression analysis was utilized. After adjusting the penalty parameter through ten-fold cross-validation, a total of 8 genes were chosen (**Figures 4C, D**). These genes encompassed F13A1, FOLR2, SCPEP1, CAMK4, KLRF1, PTGDR, SLC37A2 and BCL11B. A multivariate Cox regression model was established by including the genes identified through LASSO regression. According to the analysis, survival could be predicted independently by CAMK4, SLC37A2, and BCL11B. The risk score formula, derived from the coefficients of multivariate Cox regression, was calculated as follows: CAMK4 multiplied by 0.473431547722129, plus SLC37A2 multiplied by 0.224072082639641, plus BCL11B multiplied by -0.468973448999082 (**Figure 4E**). Moreover, the risk chart indicated that patients with gastric cancer were categorized into groups with low and high risks, according to the median risk score.

### Validating the risk model in a cohort of gastric cancer

**Figure 5:**
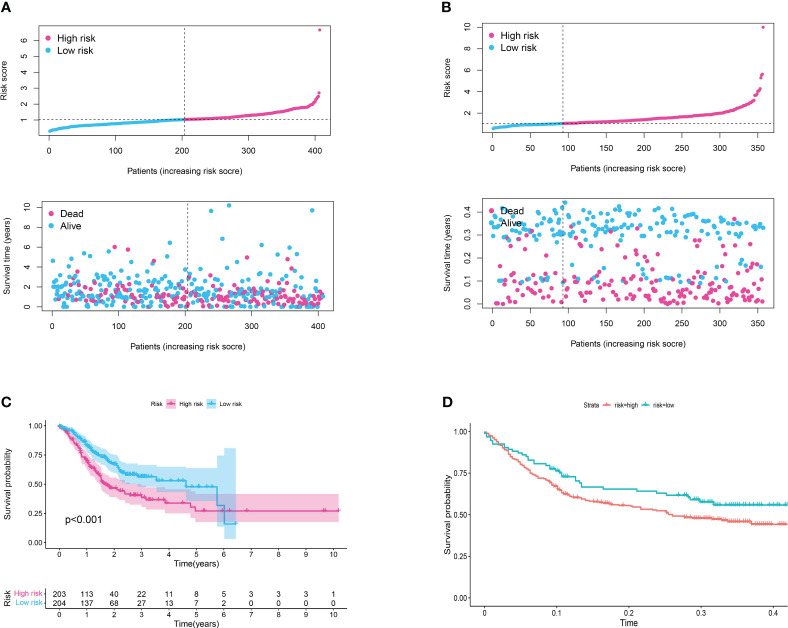
**(A)** The risk plot in TCGA cohort; **(B)** The risk plot in GEO cohort; **(C)** The survival analysis between patients involved in low- and high-risk groups of TCGA cohort; **(D)** The survival analysis between patients involved in low- and high-risk groups of GEO cohort.

**Figure 6:**
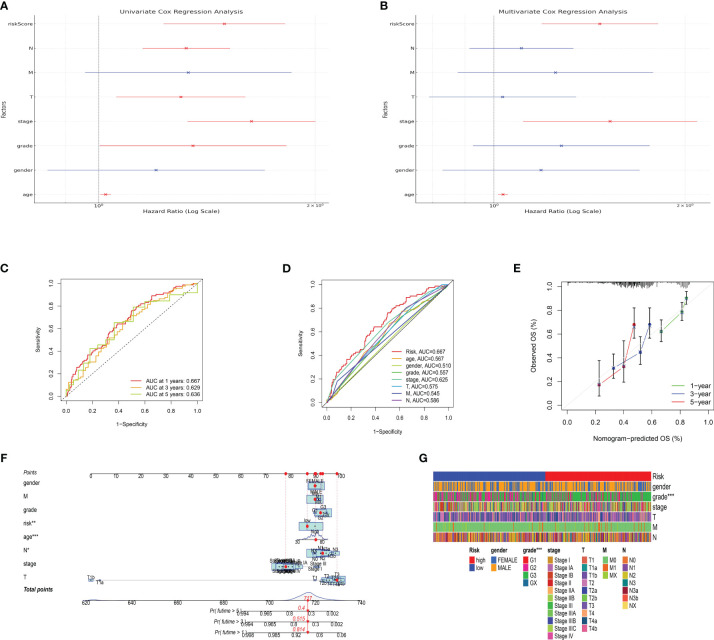
**(A)** The results of univariate independent prognosis analysis; **(B)** The results of multivariate independent prognosis analysis; **(C)** The time-dependent ROC curve revealed the AUC score of 1-year, 3-year and 5-year; **(D)** The ROC curve showed the AUC score in risk plot and clinical features; **(E)** The calibration curve showed the predictive value of nomogram; **(F)** The nomogram based on risk plot and clinical features; **(G)** The correlation analysis between clinical features and risk score.

The development and validation of a reliable risk model is of paramount importance in cancer prognosis, offering clinicians a tool to stratify patients, inform treatment decisions, and anticipate clinical outcomes. Furthermore, in order to further evaluate the predictive value of risk model in both training set and test set, the risk plot was constructed. The risk plot showed that gastric cancer patients were divided into low- and high-risk groups based on the median risk score (**Figures 5A, B**). Also, the survival analysis revealed that gastric cancer patients involved in high-risk groups showed poorer overall survival in both training and test set (**Figures 5C, D**). Moreover, to investigate the predictive significance of the risk model in a cohort of gastric cancer, we subsequently conducted multiple analyses. Furthermore, the analysis of prognostic factors independently demonstrated that age, grade, stage, T stage, N stage, and risk score are significant risk factors. Nevertheless, in the analysis of prognostic factors that are not dependent on each other, the findings indicated that the age, stage, and risk score are significant factors contributing to the risk of gastric cancer in patients (**Figures 6A, B**). To further assess the predictive significance of the risk model, we proceeded with the ROC curve analysis. The ROC curve, which varies with time, showed that the AUC scores for the 1-year, 3-year, and 5-year periods were 0.667, 0.629, and 0.636, correspondingly (**Figure 6C**). Furthermore, the clinical receiver operating characteristic (ROC) curve indicated that the risk model exhibited superior predictive efficacy compared to clinical characteristics including age, gender, grade, stage, T stage, M stage, and N stage (**Figure 6D**). Ultimately, to acquire the risk model that offers the most accurate predictions, we conducted the nomogram (**Figure 6F**). According to the calibration curve, the nomogram demonstrated enhanced ability in forecasting the prognosis of individuals with gastric cancer (**Figure 6E**). Furthermore, the correlation analysis revealed a significant association between the risk model and the grade of gastric cancer patients (**Figure 6G**).

### The risk assessment model demonstrated a strong association with immune-related characteristics in the cohort of individuals with gastric cancer

**Figure 7:**
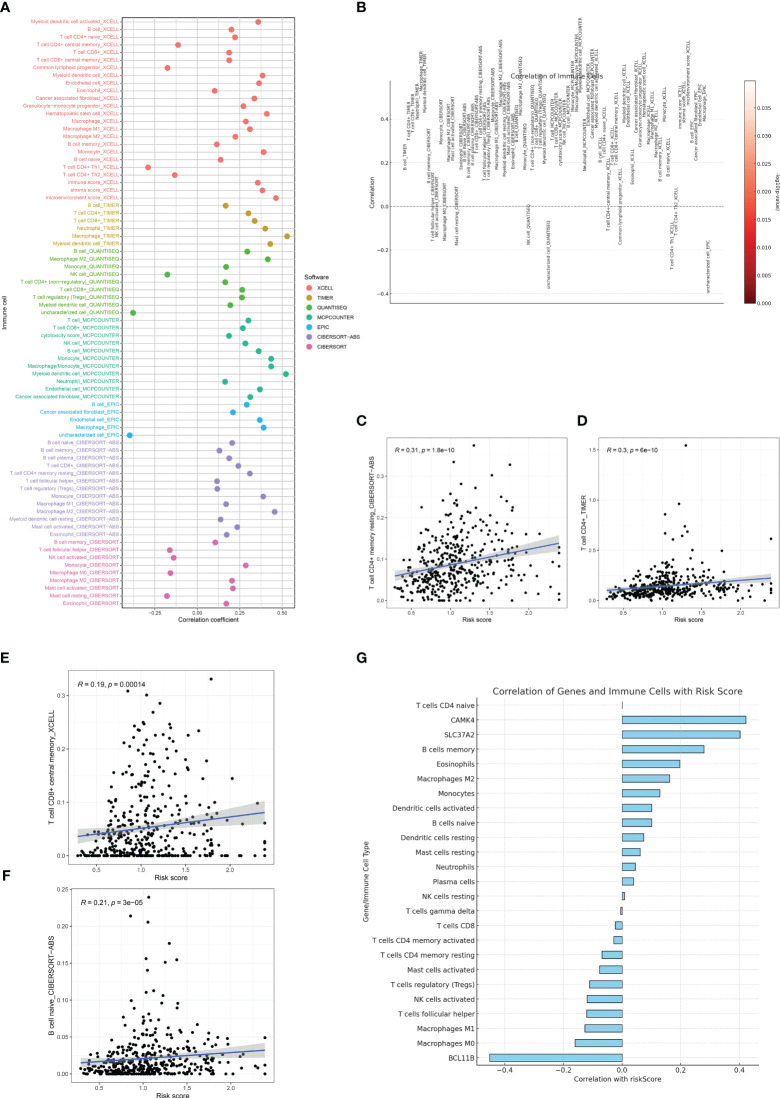
**(A, B)** The immune cell infiltration analysis based on the multiple algorithms; **(C)** The correlation between risk score and CD4+ memory T cells; **(D)** The correlation between risk score and CD4+ T cells; **(E)** The correlation between risk score and central memory CD8+ T cell; **(F)** The correlation between risk score and naïve B cell; **(G)** The correlation between risk score and immune-related cells and genes involved in risk score.

The tumor microenvironment (TME) is a complex milieu, comprising not only of tumor cells but also a myriad of stromal and immune cells that play pivotal roles in tumor progression, immune evasion, and therapy response. In order to clarify the connection between the risk score that was previously determined and the tumor microenvironment, we examined the association between the risk score and the infiltration of immune cells in samples of gastric cancer. The immune cell composition was estimated using several state-of-the-art algorithms, including CIBERSORT, quanTIseq, TIMER, and xCell.Consistently, in all algorithms, an elevated risk score was linked to heightened infiltration of macrophages and T cells (**Figures 7A–G**).

### The GSEA revealed the key pathways involved in risk model

**Figure 8:**
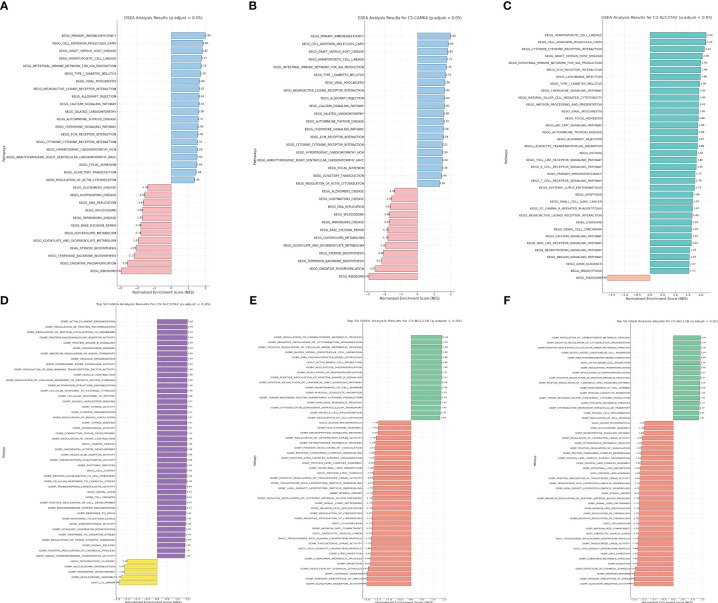
**(A)** The GSEA analysis of KEGG terms with CAMK4; **(B)** The GSEA analysis of GO terms with CAMK4; **(C)** The GSEA analysis of KEGG terms with SLC37A2; **(D)** The GSEA analysis of GO terms with SLC37A2; **(E)** The GSEA analysis of KEGG terms with BCL11B; **(F)** The GSEA analysis of GO terms with BCL11B.

Genes do not operate in isolation. They interact in networks and pathways, orchestrating a symphony of cellular processes and responses. In the context of cancer prognosis, understanding the functional implications of key prognostic genes can offer profound insights into the underlying biological mechanisms that drive patient outcomes. In order to obtain understanding into the biological pathways and processes affected by the genes included in our prognostic risk score, we conducted Gene Set Enrichment Analysis (GSEA) on the trio of genes comprised in the score. Through GSEA analysis, it was discovered that the genes associated with the risk model exhibited strong associations with numerous immune-related pathways, including primary immunodeficiency and the intestinal immune network for IGA production (**Figures 8A–F**).

### The IHC assay reveals the different distribution of immune-related markers between normal and tumor samples

**Figure 9:**
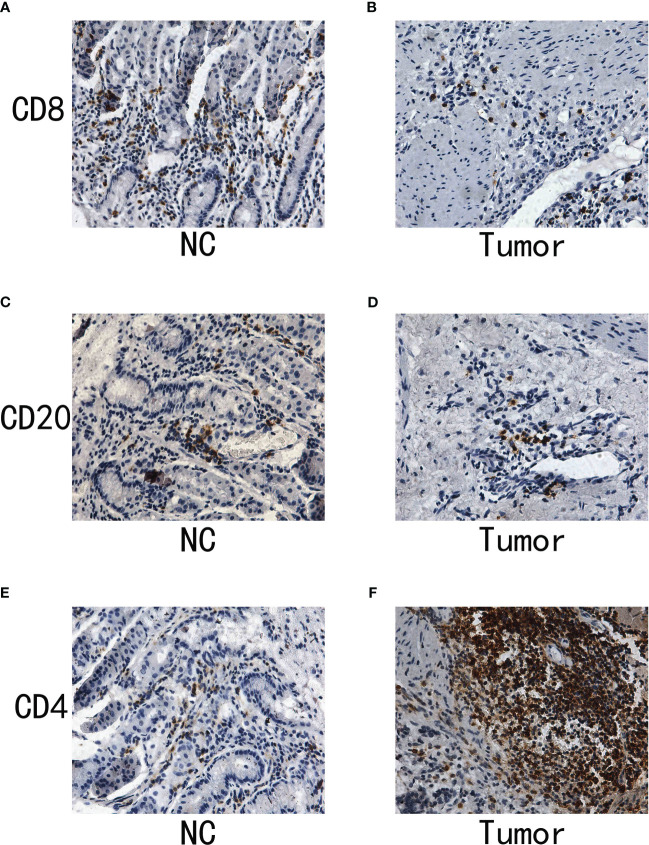
**(A)** The IHC analysis of CD8 in normal samples; **(B)** The IHC analysis of CD8 in gastric cancer samples; **(C)** The IHC of CD20 in normal samples; **(D)** The IHC of CD20 in gastric cancer samples; **(E)** The IHC of CD4 in normal samples; **(F)** The IHC of CD4 in gastric cancer samples.

Immunohistochemistry (IHC) stands as a potent tool in bridging the gap between molecular insights and the clinical reality of cancer. It provides a visual snapshot of the cellular landscape within tumor tissues, offering an opportunity to explore the presence and distribution of specific immune cell markers. In our quest to decode the immune dynamics within gastric cancer, we turned to IHC to assess the expression patterns of critical immune markers, specifically CD4, CD8, and CD20, which are pivotal in delineating T cell and B cell populations. These markers not only illuminate the immune composition within the tumor microenvironment but also hint at potential immune responses and therapeutic avenues. To demonstrate the expression pattern of immune-related markers in normal and tumor samples, we conducted the IHC assay as part of this study. The CD20 is the biomarker for B cell. In addition, the CD4 and CD20 are the biomarker for T cell. The results of immunohistochemistry (IHC) indicated that there was no notable disparity in the expression levels of CD8 and CD20 between the normal and tumor specimens (**Figures 9A–D**). In our specimens, CD4-positive T cells were predominantly identified within the lymphoid follicles and in the inter-follicular regions (**Figures 9E, F**). The results of the IHC analysis showed that the level of CD4 expression in the tumor sample is elevated compared to the normal sample.

## Discussion

The detailed examination emphasizes the complex connection among immune infiltration, patterns of gene expression, and the clinical results in individuals with gastric cancer, specifically those experiencing cachexia.

Analyzing the GEO dataset allows for the identification of genes that are expressed differently, leading to a fundamental comprehension of the molecular variances that form the basis of cachexia in individuals with gastric cancer. The considerable amount of modified genes in visceral and subcutaneous adipose tissue in cachectic individuals compared to controls highlights the extensive molecular alterations that happen in response to the advancement of cancer. This observation supports the notion that cachexia is not merely a symptom of advanced cancer but might be driven by specific molecular pathways that exacerbate disease progression.

The analysis of enriched pathways, combined with the findings from WGCNA, reveals an altered immune microenvironment in gastric cancer. The potential role of adaptive immunity in shaping the tumor microenvironment is emphasized by the favorable connections between particular gene modules and immune cells, including memory B cells and plasma cells. This is especially important when considering the emerging immunotherapies that utilize the body’s natural defense system to fight against cancer.

The prognostic model we developed, which relies on genes such as CAMK4, SLC37A2, and BCL11B, offers a hopeful approach to categorize patients according to their risk of survival. The validity of this model, as evidenced by its predictive accuracy and ability to outperform traditional clinical parameters, suggests its potential clinical utility. The correlation between this risk model and clinical characteristics, specifically grade, suggests its wider significance in comprehending the advancement and seriousness of the disease.

Moreover, the significant connection between our risk assessment and the infiltration of immune cells, particularly macrophages and T cells, provides a more profound understanding of the tumor microenvironment. Tumor growth, angiogenesis, and metastasis have been linked to the involvement of macrophages, especially tumor-associated macrophages. The heightened occurrence among patients at high risk could suggest a heightened immunosuppressive and tumor-promoting setting. The increased infiltration of T cells in high-risk patients is a subject of great interest due to their dual function as effector cells that target tumors and as regulatory cells that suppress anti-tumor immunity. Further research is necessary to explore the potential influence of various immune cells on patient outcomes and their dynamic interaction.

Lastly, the GSEA results emphasize the potential pathways through which our risk score-associated genes might influence disease progression. The connection to pathways related to the immune system strengthens the pivotal role of immunity in the development and advancement of gastric cancer.

To summarize, our research offers a diverse viewpoint on how genes and the immune system influence the clinical results of individuals with gastric cancer. The risk model that has been identified shows a promising potential for future research due to its strong association with immune infiltration and possible molecular pathways. As we move toward more personalized medicine, such models can be instrumental in guiding treatment decisions and tailoring therapeutic strategies. Future studies should focus on validating these findings in larger cohorts and exploring potential therapeutic targets within the identified pathways.

## Data availability statement

The original contributions presented in the study are included in the article/supplementary material. Further inquiries can be directed to the corresponding author.

## Author contributions

XS: Conceptualization, Investigation, Supervision, Writing – review & editing. GW: Formal Analysis, Project administration, Writing – review & editing.
